# A Theoretical Study of the Electron–Surface Optical Phonon Interaction in Monolayer Transition Metal Dichalcogenides Deposited on SiC and hexagonal BN Dielectric Substrates in the Vicinity of the Points *K*_+_(*K*_−_) of the Brillouin Zone

**DOI:** 10.3390/ma17225552

**Published:** 2024-11-14

**Authors:** Mounira Mahdouani, Ramzi Bourguiga, Spiros Gardelis

**Affiliations:** 1 Laboratoire de Physique des Matériaux Structure et Propriétés (LR01ES15), Groupe Physique des Composants et Dispositifs Nanométriques, Faculté des Sciences de Bizerte, Université de Carthage, Jarzouna-Bizerte 7021, Tunisia; mannoumah@yahoo.fr (M.M.); ramzi.bourguiga@fsb.ucar.tn (R.B.); 2 Condensed Matter Physics Section, Physics Department, National and Kapodistrian University of Athens, Panepistimiopolis, 15784 Athens, Greece

**Keywords:** transition metal dichalcogenides, polaron, surface optical phonon, polaronic oscillator strength, polaronic scattering rate

## Abstract

We theoretically investigated the electron–surface optical phonon interaction across the long-range Fröhlich coupling in monolayer transition metal dichalcogenides, such as WS_2_, WSe_2_, MoS_2_, and MoSe_2_ monolayers, on SiC and hexagonal BN dielectric substrates. We employed the effective Hamiltonian in the ${K_{+}(K}_{-})$ valley of the hexagonal Brillouin zone to assess the electronic energy shifts induced by the interaction between electronic states and surface polar optical phonons. Our results indicate that the interaction between electrons and surface optical phonons depends upon the polar nature of the substrate. We have also calculated the polaronic oscillator strength, as well as the polaronic scattering rate of the lower polaron state in monolayer WS_2_, WSe_2_, MoS_2_, and MoSe_2_ on SiC and hexagonal BN dielectric substrates. As a result, we have theoretically proved the following: firstly, the enhancement of the polaronic scattering rate with temperature, and secondly, the notable influence of the careful selection of surrounding dielectrics on both the polaronic oscillator strength and the polaronic scattering rate. Thus, optimal dielectrics would be those exhibiting both elevated optical phonon energy and a high static dielectric constant.

## 1. Introduction

In the past few years, there has been a growing interest in transition metal dichalcogenides (TMDCs), specifically in the form of monolayer (ML) van der Waals materials. This has sparked significant research interest in various applications in electronics and optoelectronics [1,2,3].

In comparison with conventional semiconductors such as GaAs, the Coulomb interaction between conduction electrons and valence holes, as well as the oscillator strengths of excitons in ML TMDCs, is significantly higher due to the two-dimensional confinement of charge carriers, heavy effective masses, and weak screening in 2D systems [4,5,6,7,8,9]. That is why scientists studying the physics of semiconductor nanosystems have been very interested in 2D materials during the past few years, such as graphene, hexagonal boron nitride MLs, and TMDC MLs, and the heterostructures they generate [10,11]. The ML TMDCs that have been investigated the most are MoS_2_, MoSe_2_, WS_2_, and WSe_2_ [12,13].

The transport of carriers in ML 2D materials under low fields is influenced by multiple scattering mechanisms, including interactions with acoustic and optical phonons. Additionally, scattering can occur due to polar coupling with the substrate beneath or with dielectrics, introducing another factor involving remote optical phonons. Thus, polar optical phonons, situated at the interface, play a significant role in scattering carriers in TMDCs through Fröhlich coupling [14,15,16,17]. Therefore, it is crucial to comprehend these scattering events by examining the coupling between surface optical phonons (SOPs) and electrons in TMDCs. Developing models that can elucidate experimental results becomes essential. This coupling is typically characterized by interactions between electronic excitations and phonons, giving rise to various intriguing effects on a crystal, including the formation of polarons [14,15,16,17,18,19,20].

Several studies in the literature proved the importance of the role played by SOP coupling in the optical properties of ML TMDCs deposited on polar substrates. As an example, we reference Suvodeep Paul et al. [21], who showed that the creation of a $WS_{2}/hBN$ heterostructure leads to the coupling between electrons in $WS_{2}$ and polar phonons in hBN. This coupling governs the enhancement of defect-bound excitons and biexcitons. Additionally, they have performed an extensive resonant Raman analysis, varying both the polarization and magnetic field, which provided further confirmation of the electron–phonon coupling in the $WS_{2}/hBN$ heterostructure.

Likewise, Colin M. Chow et al. [22] observed a resonant Raman scattering effect through cross-material exciton–phonon coupling at van der Waals interfaces. They noted that the sensitivity of excitons in monolayer materials to their phononic environments, such as those provided by $SiO_{2}$, hBN, and sapphire, can be exploited to deepen our understanding of atomically thin devices. Elsewhere, Bastian Miller et al. [23] investigated exciton–phonon coupling in charge-tunable single-layer MoS_2_ devices using polarization-resolved Raman spectroscopy. They found a strong defect-mediated coupling between the long-range oscillating electric field of the longitudinal optical phonon in the dipolar medium and the exciton.

Sanjay Gopalan et al. [24] also explored the impact of the dielectric environment on electronic transport in monolayer TMDCs. By employing ab initio methods, they calculated the low-field carrier mobility in free-standing layers, considering the effects of dielectric screening on electron–phonon interactions induced by the bottom oxide and gate insulator, as well as scattering from hybrid interface optical phonon/plasmon excitations. Their findings revealed that using insulators with a high dielectric constant can greatly improve carrier mobility.

All of these demonstrate how an understanding of the substrate-dependent SOP coupling provides a foundation for tailoring and enhancing the optical and electronic properties of ML TMDC-based devices, guiding both material and substrate selection for specific device functionalities.

This paper is structured as follows: First, we theoretically investigate the interaction between electrons and surface optical phonons in ML TMDCs on polar substrates, such as silicon carbide (SiC) and hexagonal boron nitride (hBN). Furthermore, we present a theoretical examination of the polaronic oscillator strength in ML TMDCs on polar substrates. Finally, we investigate the temperature dependence of the polaronic scattering rate in ML TMDCs on SiC and hBN polar substrates.

## 2. Electron–Surface Optical Phonon Interaction in ML TMDCs on SiC and hBN Dielectric Substrates

**Figure 1:**
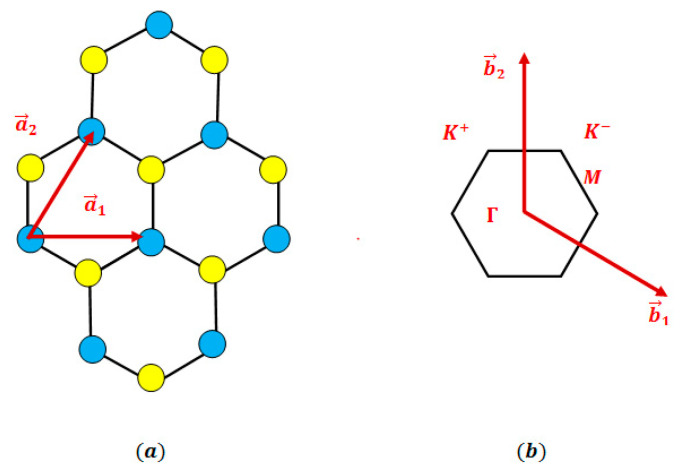
(**a**) The triangular Bravais lattice of monolayer transition metal dichalcogenides MX_2_. Blue and yellow full circles denote the metal (M) and chalcogenide (*X*) atoms, respectively. (**b**) The first Brillouin zone and high-symmetry points Γ, K, and M of the TMDCs in the reciprocal space of the triangular lattice. Its primitive lattice vectors are ${\vec{b}}_{1}$ and ${\vec{b}}_{2}$.

A monolayer transition metal dichalcogenide (TMDC) consists of a central layer of metal M atoms arranged in a triangular lattice, flanked by two layers of chalcogen X atoms positioned on the same triangular lattice. The triangular Bravais lattice is defined by the following basis vectors:

${\vec{a}}_{1}=a_{0}\left(1,0,0\right)$ and ${\vec{a}}_{2}=\frac{a_{0}}{2}\left(1,\sqrt{3},0\right)$ (see Figure 1a).

Figure 1b illustrates the reciprocal lattice, defined in relation to the triangular Bravais lattice and characterized by the following vectors:

${\vec{b}}_{1}=\frac{4\pi }{\sqrt{3}a_{0}}\left(\frac{\sqrt{3}}{2},-\frac{1}{2},0\right)$ and ${\vec{b}}_{2}=\frac{4\pi }{\sqrt{3}a_{0}}\left(0,1,0\right)$, where $a_{0}$ is the lattice constant.

The two-dimensional Brillouin zone of the TMDCs exhibits a hexagonal shape, featuring high-symmetry points denoted as Γ,  K, and M, each defined as follows:

$$\Gamma =\left(0,\;0\right),\;K=\left(\frac{2\pi }{3a_{0}},\;\frac{-2\pi }{\sqrt{3}a_{0}}\right),\;M=\left(\frac{\pi }{a_{0}},\;\frac{\pi }{\sqrt{3}a_{0}}\right)$$

The effective 2 × 2 Hamiltonian characterizing the states of the conduction and valence bands with the parallel spins $s=+\frac{1}{2}$ in the vicinity of the point $K_{+}$ is represented by the following expression [25,26,27]:

(1) $$H_{+}=\left(\begin{array}{cc} \frac{E_{g}}{2} & \gamma \left(k_{x}-ik_{y}\right)\\ \gamma \left(k_{x}+ik_{y}\right) & -\frac{E_{g}}{2} \end{array}\right)$$

where $\;k=\left(k_{x},\;k_{y}\right)$ denotes the two-dimensional wave vector of the electrons measured from the point $K_{+}$; the parameter γ is directly proportional to the interband matrix element of the momentum operator $\gamma =\frac{P^{2}}{m^{*}}$, where $m^{*}$ is the electron effective mass; and $E_{g}$ represents the width of the band gap.

The Hamiltonian describing a pair of spin sub-levels with $s=-\frac{1}{2}$ in the same valley has the form of Equation (1) through the substitution $E_{g}\to E_{g}+∆$, where ∆ represents the sum of the spin–orbit splitting of the conduction and valence bands. The effective Hamiltonian in the $K_{-}$ valley is derived from Equation (1) through the substitution $\left(k_{x}\pm ik_{y}\right)\to \left(k_{x}\mp ik_{y}\right)$.

The energy spectrum of the electrons derived from the Hamiltonian in Equation (1) has the following Dirac form:

(2) $$\varepsilon_{\lambda ,k}=\lambda \varepsilon_{k}=\sqrt{{\left(\frac{E_{g}}{2}\right)}^{2}+\gamma^{2}k^{2}}$$

Here, λ=+ and λ=− correspond to the conduction and valence bands, respectively.

In our study, we use the assumption of homogeneous and defect-free interfaces between transition metal dichalcogenide monolayers and dielectric substrates as a simplification often used in theoretical models and simulations to make the problem tractable.

In this work we have investigated the electron–surface optical phonon (SOP) interaction in ML TMDCs on SiC and hexagonalBN dielectric substrates across long-range Fröhlich coupling. Indeed, the long-range Fröhlich coupling model provides a robust framework for understanding electron–SOP interactions in TMDCs on polar substrates. However, it relies on several approximations, for example the Born–Oppenheimer Approximation [28]. Short-range interactions [29] (e.g., electron–phonon interactions in non-polar materials) are not considered. A constant effective mass for the electron is assumed, whereas non-linear interactions and multi-phonon processes are typically neglected [30]. Impurities, defects, and other forms of disorder that can affect the electron–phonon interaction in real materials are usually not included in the idealized Fröhlich model [28,31]. Phonon dispersion is typically assumed to be linear, which is an approximation that might not hold for all phonon modes or substrates [32]. Often, a single dominant phonon mode is considered, neglecting the possible contribution of multiple phonon modes [29].

To simplify our analysis, we consider the phonon spectrum as isotropic, implying that phonons exhibit either longitudinal or transverse polarization. The Fröhlich Hamiltonian introduces an interaction term wherein an electron scatters from $\vec{k}$ to ${\vec{k}}^{\prime }=\vec{k+q}$, involving the emission or absorption of a phonon. In both cases, the total momentum is conserved and is expressed as follows:

(3) $${H=H}_{ph}+H_{e-ph}$$

The term ${\;H}_{ph}$ denotes the phonon energies, incorporating both the longitudinal optical (LO) and surface optical (SO) modes, and can be expressed as follows:

(4) $$H_{ph}=\sum_{q,\nu }\hbar \omega_{\nu }{\;a}_{q}^{+}a_{q}$$

In this context, ${\;a}_{q}^{+}$ and $a_{q}$ represent the creation and annihilation operators, respectively, for the phonon characterized by the wave vector q, while $\omega_{\nu }$ refers to the frequency of the phonon.

The second term $H_{e-ph}$ is the Hamiltonian of the interaction between the electron and phonon [33]:

(5) $$H_{e-ph}=\sum_{q,\nu }M_{q,\;\;\nu }\left({\;a}_{-q}^{+}{+a}_{q}\right){\;e}^{-iq\;r}$$

The Fröhlich Hamiltonian is given as follows:

(6) $$H=\sum_{q,\nu }\hbar \omega_{\nu }{\;a}_{q}^{+}a_{q}+\sum_{q,\nu }M_{q,\;\;\nu }\left({\;a}_{-q}^{+}{+a}_{q}\right){\;e}^{-iq\;r}\;\;\;$$

The interaction between carriers in monolayer transition metal dichalcogenides (TMDCs) and surface optical phonons is described by the second term in Equation (6).

The coupling element in the Fröhlich Hamiltonian $M_{q,\;\nu }$ represents the interaction between the electron in TMDCs and the surface optical phonon of the polar substrates. This matrix element is expressed as follows [34,35,36]:

(7) $$V_{SOP}{=M}_{q,SO}=\left|\vec{k}-\vec{k+q}\right|\sqrt{\frac{e^{2}\;F_{\nu }^{2}}{2NAq}}e^{-qz_{0}}$$

In the given context, $F_{\nu }^{2}$ represents the magnitude of the polarization field, which is determined by the Fröhlich coupling [37]:

(8) $$F_{\nu }^{2}=\frac{\hbar \omega_{SO,\nu }}{2\pi }\left(\frac{1}{\varepsilon_{\infty }+\varepsilon_{env}}-\frac{1}{\varepsilon_{0}+\varepsilon_{env}}\right)$$

where ${\;\varepsilon }_{0}$ and $\varepsilon_{\infty }$ are the low- and high-frequency dielectric constants of the polar substrate (refer to Table 1), and $z_{0}$ represents the internal distance between the TMDCs and the polar substrate (refer to Table 2). The term $\hbar \omega_{SO,\nu }$ denotes the energy of the SO phonons of the polar substrates with two branches, ν=1, 2.

The SOP energies are extracted from the bulk longitudinal optical (LO) phonons as follows [38]:

(9) $$\hbar \omega_{SO}=\hbar \omega_{LO}{\left(\frac{1+\frac{1}{\varepsilon_{0}}}{1+\frac{1}{\varepsilon_{\infty }}}\right)}^{\frac{1}{2}}$$

The screening of the Coulomb interaction by the polar dielectric environment is considered through ${\;\varepsilon }_{env}$. Given the weak screening of the electric field perpendicular to the plane of the ML TMDCs, ${\;\varepsilon }_{env}\;$ is set to 1 [44].

On polar substrates, surface optical phonons (SOPs) induce an electric field that interacts with the electrons in the neighboring ML TMDCs. Using Equations (7) and (8), the SOP coupling is expressed as

(10) $${W=\sum_{\vec{q}}\left|\left\langle \psi_{k}|V_{SOP}|\psi_{k+q}\right\rangle \right|}^{2}=\frac{NA}{{\left(2\pi \right)}^{2}}\iint \frac{1-\cos\left(\theta_{k}-\theta_{k+q}\right)}{2}\frac{4\pi^{2}e^{2}F_{\nu }^{2}}{NAq}e^{-2qz_{0}}qdqd\theta_{q}.$$

The summation is performed over one spin and one valley, where $A=\frac{\sqrt{3\;}}{2}a^{2}$ is the area of the two-atom unit cell.

In our analysis in the present case, we have followed the same theoretical method presented in our previous calculations [14,15,16]. So, to study the interactions between electrons and surface optical phonons in ML TMDCs, we have specifically considered the electronic states $\left|\psi_{k\;}\rangle \right.$ and $\left|\psi_{k+q\;}\rangle \right.$, with electron energies $E_{k}=\varepsilon_{k}$ and $E_{k+q}=\varepsilon_{k+q}$, respectively. We have also considered the effective 2 × 2 Hamiltonian characterizing the states of the conduction and valence bands with the parallel spins $s=\pm \frac{1}{2}$ in the vicinity of the point ${K_{+}(K}_{-})$ of the hexagonal Brillouin zone.

The space of polaronic states results from a tensor product between the two subspaces of electronic and phononic states. Thus, we consider new states called polaronic states given by

(11) $$\left\{\;\left|\psi_{k+q\;\;},\;0q\rangle \right.\;,\left|\psi_{k\;},\;1q\rangle \right.\right\}.$$

The polaron electron energies $E_{\pm }^{e}$ for the states $\left|\psi_{\pm }\rangle \right.$ in ML TMDCs on polar substrates are given below [14,15,16]:

(12) $$E_{\pm }^{e}=\frac{1}{2}\left(E_{k+q}+E_{k}+\hbar \omega_{LO}\right)\pm \sqrt{{\left[\frac{1}{2}\left(E_{k+q}-E_{k}+\hbar \omega_{LO}\right)\right]}^{2}+\frac{NA}{{\left(2\pi \right)}^{2}}\iint \frac{1-\cos\left(\theta_{k}-\theta_{q}\right)}{2}\frac{4\pi^{2}e^{2}F_{\nu }^{2}}{NAq}e^{-2qz_{0}}qdqd\theta_{q}}$$

**Figure 2:**
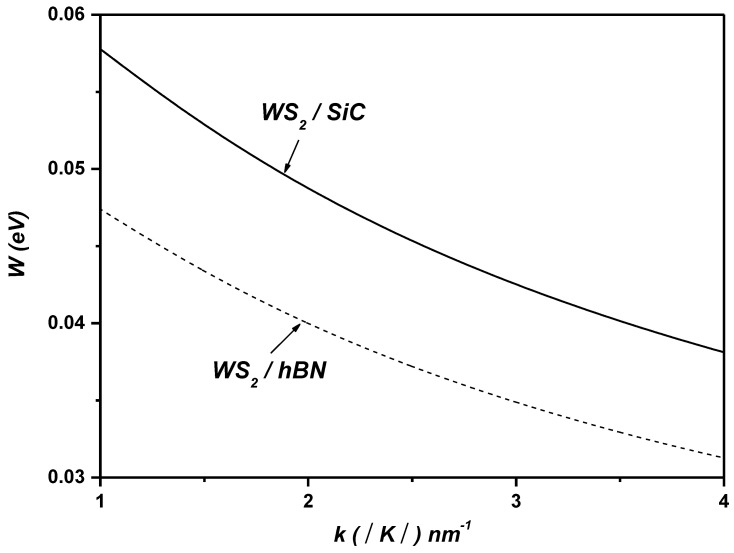
The SO coupling strength between the electronic states $\left|\psi_{k\;}\rangle \right.$ and $\left|\psi_{k+q\;}\rangle \right.$ versus the wave vector k in ML WS_2_ on SiC and hBN polar substrates.

Figure 2 depicts the SO coupling strength between the electronic states $\left|\psi_{k\;}\rangle \right.$ and $\left|\psi_{k+q\;}\rangle \right.$ versus the wave vector k in ML WS_2_ on the SiC and hBN polar substrates. As shown in Figure 2, it is evident that the coupling with SOPs is significantly influenced by the type of polar substrate.

**Figure 3:**
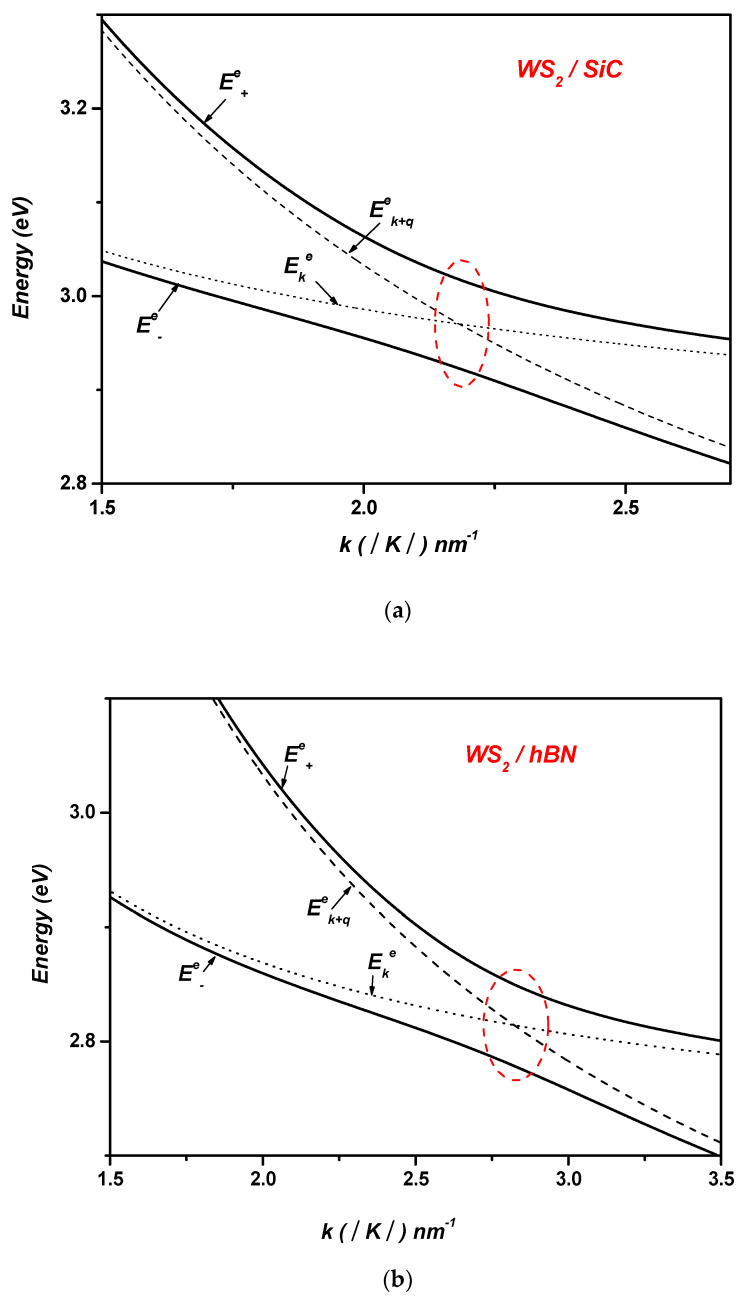

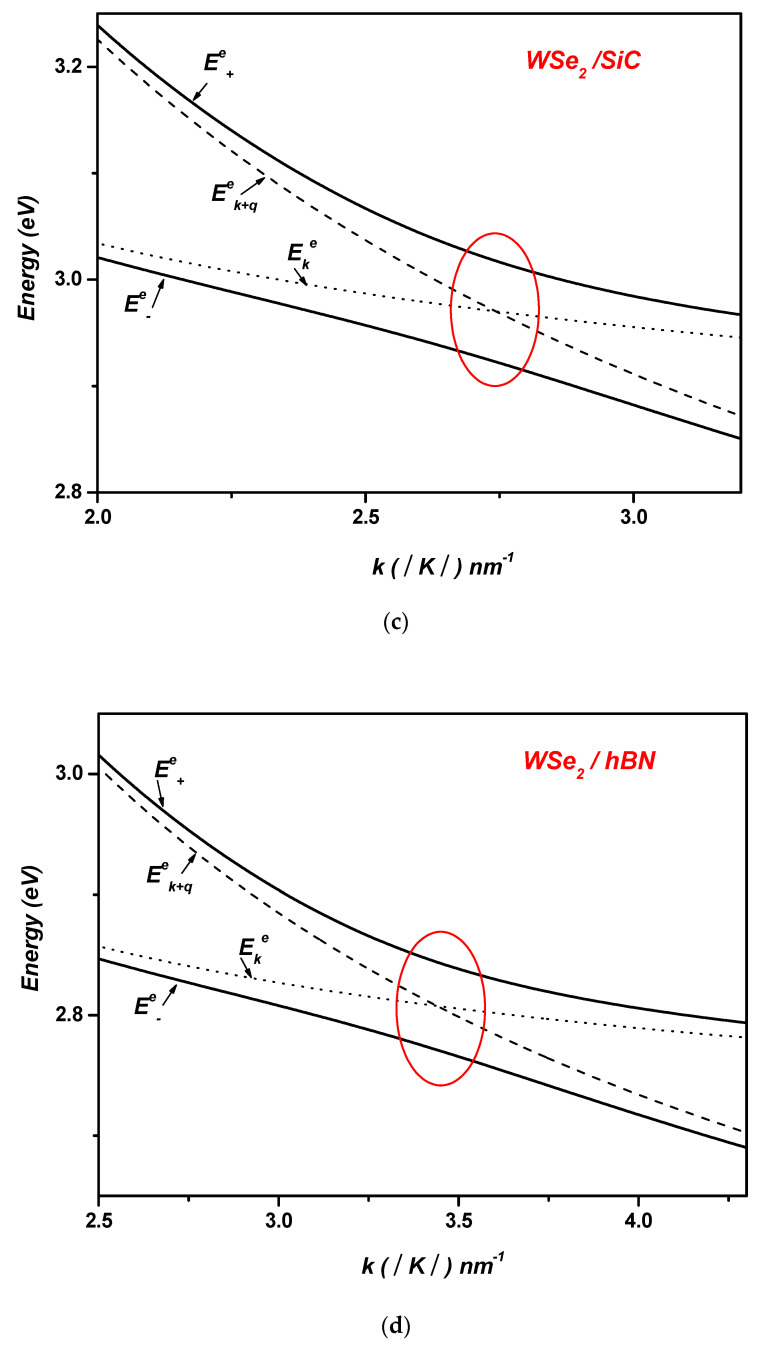

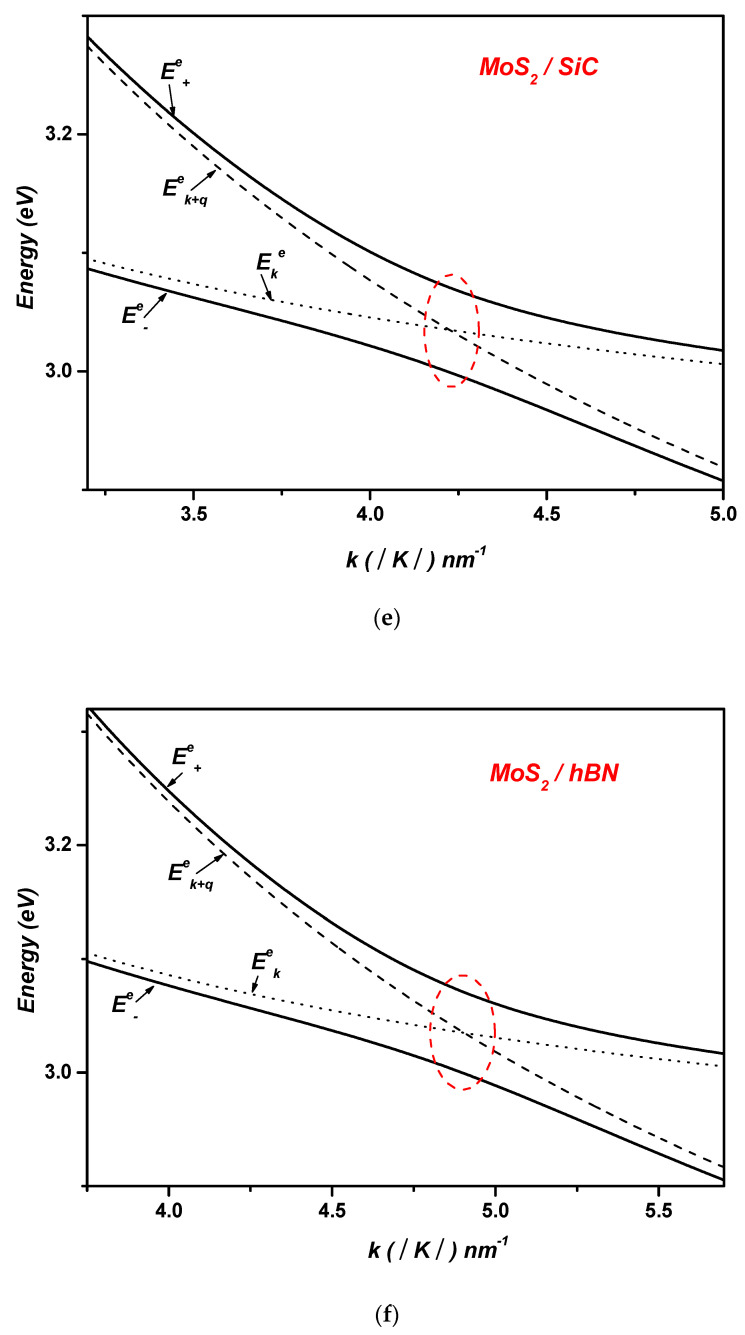
Polaron electron energies as a function of the wave vector k  in the following ML TMDCs on SiC and hBN polar substrates, respectively: (**a**) and (**b**) WS_2_; (**c**) and (**d**) WSe_2_; (**e**) and (**f**) MoS_2_.

We show in Figure 3 the polaron electron energies versus the wave vector k in ML $WS_{2},WSe_{2},MoS_{2}$ on SiC and hBN polar substrates. For comparison purposes, we have included in the same figure the energies of the noninteracting states $\left|\psi_{k+q\;},\;0q\rangle \right.\;$ and $\left|\psi_{k\;},\;1q\rangle \right.$. For example, in the case of WS_2_, these noninteracting levels cross near $k\sim 2.2\;{\;\mathrm{nm}}^{-1}$ in the case of SiC and near $k\sim 2.85{\;\mathrm{nm}}^{-1}$ in the case of hBN indicating resonant coupling (see Table 3). These crossings indicate that the separation between electronic levels is equal to $\hbar \omega_{LO}$ in both the SiC and hBN cases, where $\hbar \omega_{LO}=123.2\;\mathrm{meV}\;and\;\hbar \omega_{LO}=103.7\;\mathrm{meV}$ respectively. In fact, the electronic level crossings are clearly replaced by significant anticrossings, with energy levels approximately at ~94 meV and ~70 meV for SiC and hBN  polar substrates, respectively. In Figure 3, the enhancement of the Rabi splitting of the electron levels when shifting from hBN to SiC can be also observed (refer to Table 4, Figure 3).

In polar substrates, surface optical (SO) phonons generate an electric field that extends into the overlying monolayer. This electric field directly couples with the electrons in the TMDC layer through dipole interactions, resulting in a significant enhancement of the coupling strength. This interaction manifests as a renormalization of the electronic levels, which increases the energy splitting between the hybridized states and enhances the Rabi splitting.

The difference in Rabi splitting between SiC and hBN as polar substrates for a TMDC monolayer (see Table 4 and Figure 3) can be attributed to the distinct surface phonon properties, dielectric characteristics, and phonon energy scales of each material. SiC, for instance, has a higher dielectric constant than hBN. A higher dielectric constant typically results in stronger electric fields generated by surface phonons near the interface, thereby enhancing the coupling with the electronic states of the TMDC. This intensification of the electron–phonon interaction can lead to larger Rabi splitting.

Conversely, hBN, with its lower dielectric constant, produces relatively weaker electric fields associated with its surface phonons. Consequently, the electron–phonon coupling with the TMDC monolayer is slightly weaker, resulting in smaller Rabi splitting. This comparison underscores the critical role that the choice of polar substrate plays in determining the optical properties and performance of TMDC-based devices.

In these anticrossings, the wave functions of the levels become mixed, allowing for multiple transitions, such as $E_{k}\to E_{\pm }^{e}$, $E_{k}\to E_{k}+\hbar \omega_{LO}$, and $E_{k}\to E_{k+q}$. This demonstrates that the interaction between electrons and surface polar phonons cannot be considered a weak coupling. The coupling between electrons and SOPs leads to the Rabi splitting of the electronic levels. Hence, the calculations indicate the possibility of an energetically resonant coupling between the electronic sub-levels and the surface vibration modes in ML TMDCs on the studied polar substrates. Furthermore, the two resulting polaron states can be expressed as follows:

(13) $$\left|\psi_{\pm }\rangle \right.=\alpha_{\pm }\left|\psi_{k+q\;\;},\;0q\rangle \right.+\beta_{\pm }\left|\psi_{k\;\;},\;1q\rangle \right.\;$$

The weight of the electronic component$\;\alpha_{\pm }$ and the weight of the one-phonon component $\beta_{\pm }$ of the polaron states ± vary with the polaron energies $E_{\pm }^{e}$. The expressions detailing these dependencies are as follows [14,15,16]:

(14) $${\left|\alpha_{\pm }\right|}^{2}=\frac{{\left(E_{\pm }^{e}-\hbar \omega_{LO}\right)}^{2}}{{\left(E_{\pm }^{e}-\hbar \omega_{LO}\right)}^{2}+\frac{NA}{{\left(2\pi \right)}^{2}}\iint \frac{1-\cos\left(\theta_{k}-\theta_{k+q}\right)}{2}\frac{4\pi^{2}e^{2}F_{\nu }^{2}}{NAq}e^{-2qz_{0}}qdqd\theta_{q}}\;$$

(15) $${\left|\beta_{\pm }\right|}^{2}=\frac{\frac{NA}{{\left(2\pi \right)}^{2}}\iint \frac{1-\cos\left(\theta_{q}\right)}{2}\frac{4\pi^{2}e^{2}F_{\nu }^{2}}{NAq}e^{-2qz_{0}}qdqd\theta_{q}}{{\left(E_{\pm }^{e}-\hbar \omega_{LO}\right)}^{2}+\frac{NA}{{\left(2\pi \right)}^{2}}\iint \frac{1-\cos\left(\theta_{k}-\theta_{k+q}\right)}{2}\frac{4\pi^{2}e^{2}F_{\nu }^{2}}{NAq}e^{-2qz_{0}}qdqd\theta_{q}}$$

where $\sqrt{\frac{NA}{{\left(2\pi \right)}^{2}}\iint \frac{1-\cos\left(\theta_{k}-\theta_{k+q}\right)}{2}\frac{4\pi^{2}e^{2}F_{\nu }^{2}}{NAq}e^{-2qz_{0}}qdqd\theta_{q}}\;$ is the SO coupling strength between the electronic states $\left|\psi_{k\;},\;1q\rangle \right.\;and\;\left|\psi_{k+q\;},\;0q\rangle \right.\;$ [14,15,16,17].

**Figure 4:**
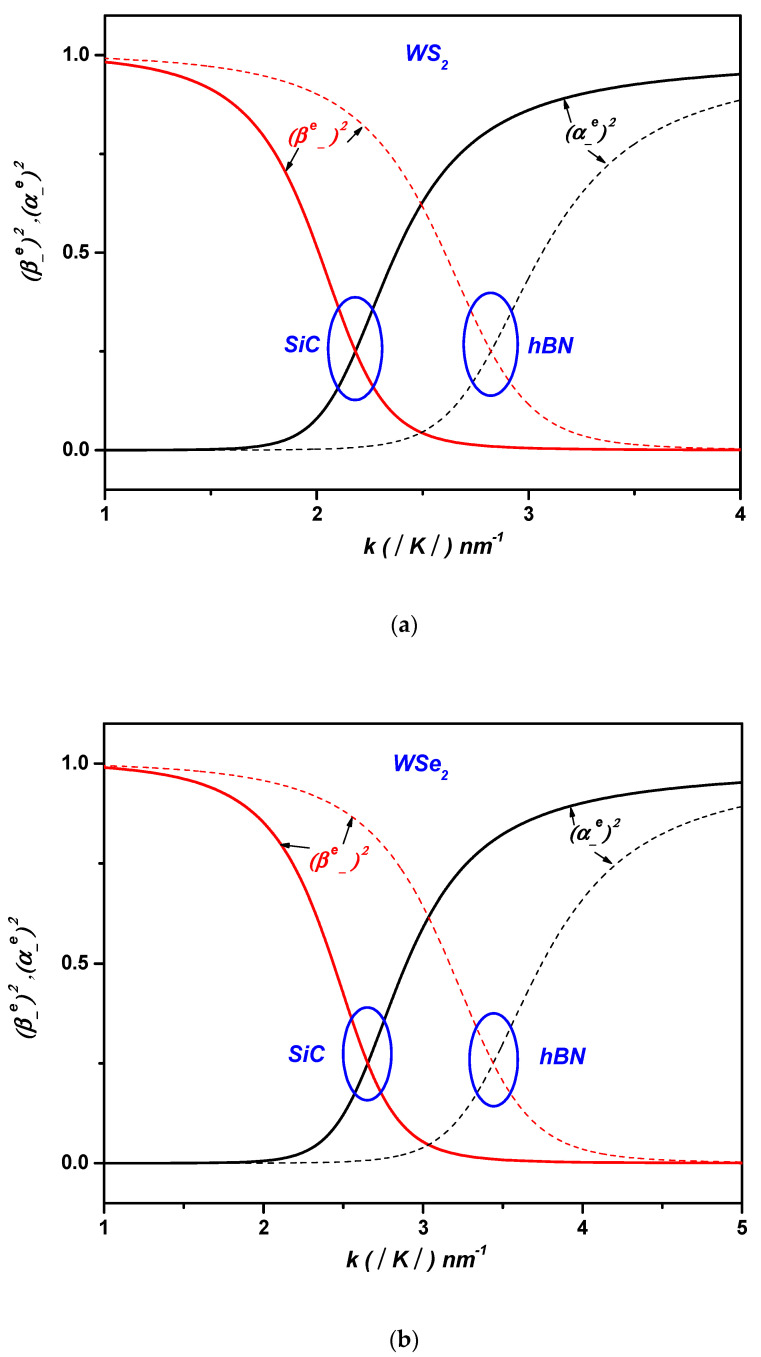

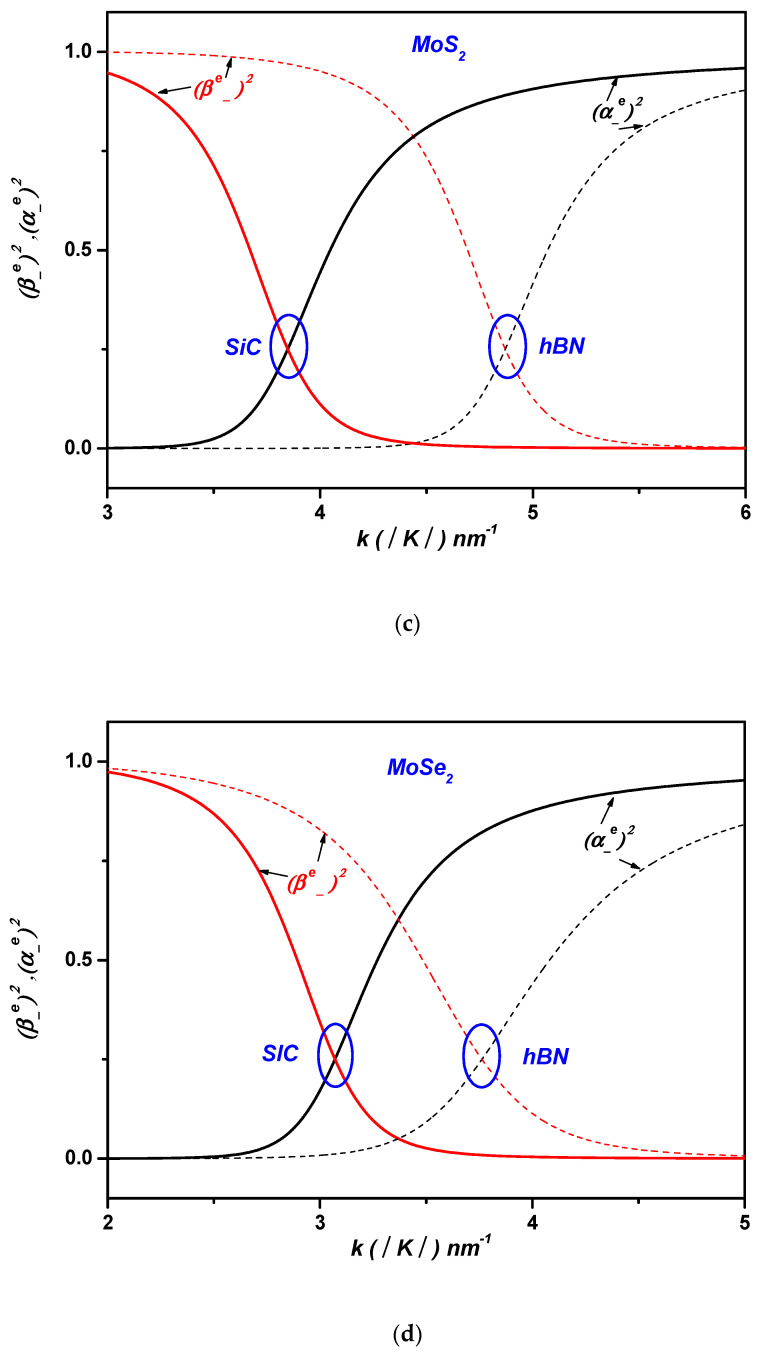
The weight of the electronic components and the one-phonon components of the lower polaron state $\left|\psi_{-}\rangle \right.$ in ML TMDCs on the SiC and hBN dielectric substrates (**a**) in $WS_{2}$, (**b**) in $W{Se}_{2}$, (**c**) in $MoS_{2}$, and (**d**) in $MoSe_{2}$ versus the wave vector k.

Figure 4 depicts the weight of the electronic components and the one-phonon components of the lower polaron state $\left|\psi_{-}\rangle \right.$ in ML TMDCs for SiC and hBN polar substrates as a function of the wave vector k in ML TMDCs. It can be seen, for example, in the case of $WS_{2}$ on the SiC polar substrate (see Table 3, Figure 4) that when the wave vector k approaches $k\sim 2.2\;\;{nm}^{-1},$ the value of the weight of the one-phonon component for the lower polaron state $\left|\psi_{-}\rangle \right.$ is much larger compared with that of the electronic component $\left(\beta_{\pm }\gg \alpha_{\pm }\right)$. This result demonstrates that the SOP situated at the interface of the ML $WS_{2}$ on the SiC polar substrate plays a crucial role in the resonant coupling between the noninteracting states $\left|\psi_{k\;},\;1q\rangle \right.$ and $\left|\psi_{k+q\;},\;0q\rangle \right.$, allowing for the formation of the polaron states. It can be noted that the same result has been proved for the other cases of ML TMDCs on the SiC and *h*BN polar substrates (see Table 3 and Figure 4).

## 3. Polaronic Oscillator Strength of ML TMDCs on SiC and hBN Polar Substrates

In the following section, we theoretically investigate the polaronic oscillator strength (OS), which is another crucial quantity. Drawing an analogy with the oscillator strengths of interband transitions in quantum dots, we have computed the OS for the interband transitions in ML TMDCs on polar substrates. In the strong confinement limit, the OS is linked to the overlap integral of the polaronic states, ${\left|\left(\psi_{-}\right)\right|}^{2}$, by the following equation [45,46]:

(16) $$f_{Osc}=\frac{{\left|\left(\psi_{-}\right)\right|}^{2}E_{p}}{2E_{PL}}$$

where $E_{P}$ is the Kane energy and $E_{PL}$ is the emission energy for one phonon of the ML TMDCs on the polar substrates, which is given by

(17) $$E_{PL}=\left(\;E_{g}+E_{-}^{e}\right),$$

where $E_{-}$ is the lower polaron energy of the exciton, $\hbar \omega_{LO}$ is the emitted photon energy, and $E_{g}$ is the energy gap of the ML TMDCs.

We have calculated the OS for the lower polaron state $\left|\psi_{-}\rangle \right.$, which is a linear combination of the two states $\left|\psi_{k+q\;},\;0q\rangle \right.$ and $\left|\psi_{k\;},\;1q\rangle \right.$:

(18) $$\left|\psi_{-}\rangle \right.=\alpha_{-}\left|\psi_{k+q\;\;},\;0q\rangle \right.+\beta_{-}\left|\psi_{k\;\;},\;1q\rangle \right.\;$$

**Figure 5:**
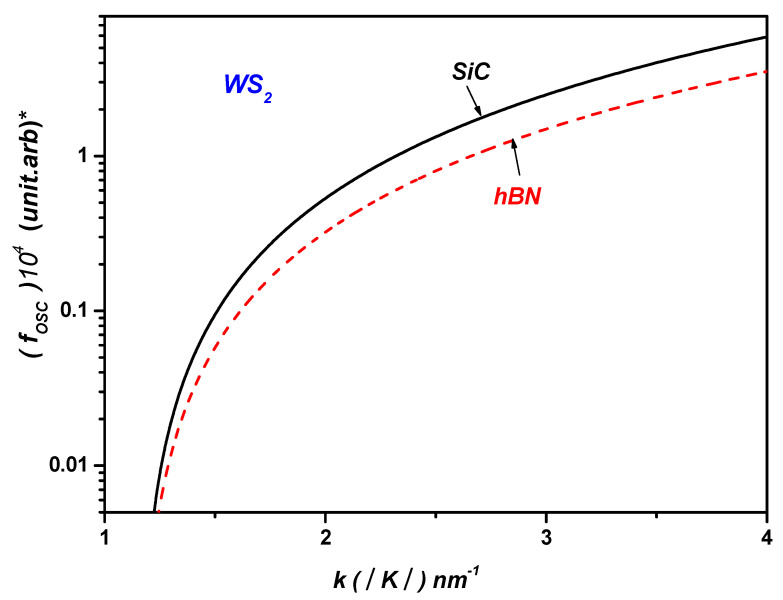
The polaronic OS of $WS_{2}$ on the SiC and hBN polar substrates versus the wave vector k in the ML $WS_{2}$. (*) in y-axis is multiplication sign.

Figure 5 shows the polaronic OS of $WS_{2}$ on the SiC and hBN polar substrates versus the wave vector k in ML $WS_{2}$.

As a result, we have theoretically proven that the polaronic OS is especially sensitive to the phonon mode of the surrounding dielectrics. In fact, this result is due to the emission energy for one phonon of the ML TMDCs for the SiC and hBN polar substrates, which is given by $E_{PL}=\left(\;\hbar \omega_{LO}=E_{g}+E_{-}\right)$. Hence, the highest polaronic oscillator strength corresponds to the highest optical phonon energy of the polar substrates, $E_{PL}=\left(\;\hbar \omega_{LO}\right)$. This is analogous to the polaronic oscillator strength in ML TMDCs, which is also much higher compared with conventional semiconductors [6,7,8,9].

Using the same method, we can easily prove that for the other ML TMDCs such as $W{Se}_{2}$*,* $MoS_{2}$, and $Mo{Se}_{2}$,

(19) $$f_{Osc}\left[MLTMDCs/SiC\right]>f_{Osc}\left[MLTMDCs/hBN\right].$$

**Figure 6:**
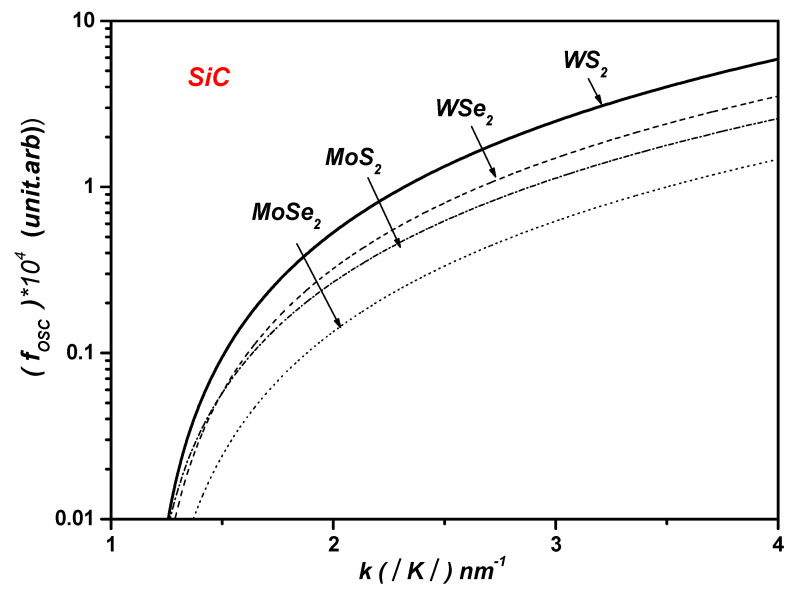
The polaronic OS in the MLs $WS_{2},WSe_{2},MoS_{2},\;\;and\;MoSe_{2}$ on the SiC polar substrate versus the wave vector k . (*) in y-axis is multiplication sign.

This result is due to the SiC dielectric constant, as well as the SiC phonon energy, compared with that of hBN $\left(\hbar \omega_{LO}\;\left(SiC\right)=123.2\;\mathrm{meV}>\hbar \omega_{LO}\;\left(hBN\right)=103.7\;\mathrm{meV}\right)$ (see Table 1), so the polarization field created near the interface should be the highest in the case of the SiC substrate compared with the hBN substrate. This result leads to the highest polaronic OS in ML TMDCs on the SiC polar substrate. Similarly, it can be concluded from Figure 6 that

(20) $$f_{Osc}\left(WS_{2}\right)>f_{Osc}\left(W{Se}_{2}\right)>f_{Osc}\left(MoS_{2}\right)>f_{Osc}\left(Mo{Se}_{2}\right).$$

Hence, this result can be explained by the difference in the electron effective masses in ML TMDCs. This result actually confirms the decrement in the polaronic OS for heavy electrons in the nearby ML TMDC/dielectric substrate interface. Otherwise, in the case of light electrons, the polaronic OS increases considerably in the strong confinement regime.

## 4. Polaronic Scattering Rate of ML TMDCs on SiC and hBN Polar Substrates

Now, we consider the temperature dependence of the polaronic scattering rate due to the SO phonons. The polaronic scattering rate (SO phonon scattering rate) is given as follows [17]:

(21) $$\frac{1}{\tau_{Polaron}}=\frac{2\pi }{\hbar }\sum_{q}{\left|M_{k,k+q}\right|}^{2}\left[1-\cos\left(\theta_{k}-\theta_{k+q}\right)\right]\times \left\{N_{q}\delta \left(E_{k}-E_{k+q}+\hbar \omega_{q}\right)+\left(N_{q}+1\right)\delta \left(E_{k}-E_{k+q}-\hbar \omega_{q}\right)\right\}$$

Here, $N_{q}$ is the Bose–Einstein phonon occupation number, $\theta_{k}\;$ is a directional angle of the wave vector $\vec{k}$, and ${\left|M_{k,k+q}\right|}^{2}$ is given by

(22) $${\left|M_{k,k+q}\right|}^{2}={\left|\left\langle \psi_{k}|V_{SOP}|\psi_{k+q}\right\rangle \right|}^{2}=\left[\frac{1-\cos\left(\theta_{k}-\theta_{k+q}\right)}{2}\frac{4\pi^{2}e^{2}F_{\nu }^{2}}{N\frac{\sqrt{3\;}}{2}a^{2}q}e^{-2qz_{0}}\right].$$

The summation ∑ is replaced by the integral $\frac{NA}{4\pi^{2}}\iint qdqd\theta$ (the sum over a spin and a valley), where $\;A=\frac{\sqrt{3}}{2}a^{2}$ is the area of the elementary cell that contains two atoms.

Thus,

(23) $$\frac{1}{\tau_{Polaron}}=\frac{2\pi }{\hbar }\frac{NA}{{\left(2\pi \right)}^{2}}\iint {\left|M_{k,k+q}\right|}^{2}\left[1-\cos\left(\theta_{k}-\theta_{k+q}\right)\right]\times \left\{N_{q}\delta \left(E_{k}-E_{k+q}+\hbar \omega_{q}\right)+\left(N_{q}+1\right)\delta \left(E_{k}-E_{k+q}-\hbar \omega_{q}\right)\right\}qdqd\theta_{q}$$

**Figure 7:**
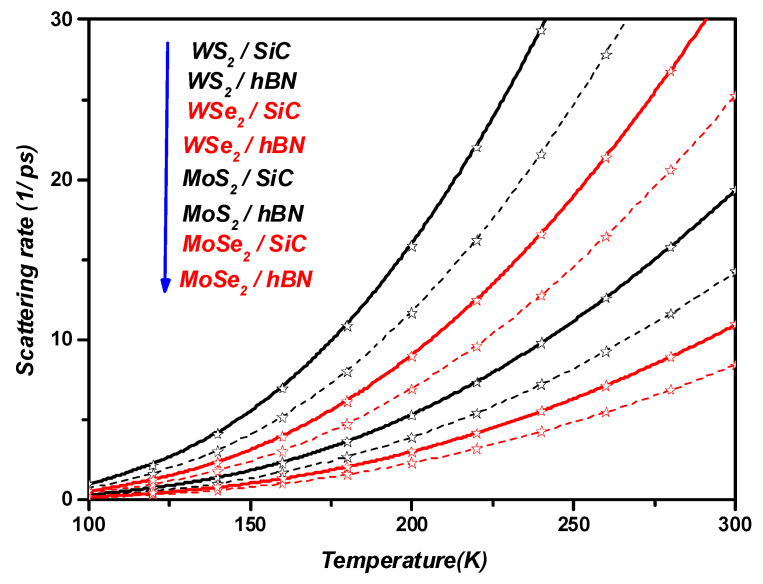
The temperature dependence of the SO phonon scattering rate in the MLs $WS_{2},WSe_{2},MoS_{2},\;and\;\;MoSe_{2}$ on the SiC and hBN polar substrates. Stars represent the calculated points on the graph.

Figure 7 shows the temperature dependence of the SO phonon scattering rate in ML TMDCs on the SiC and *h*BN polar substrates. We clearly see that at temperatures higher than room temperature, the SO phonon scattering rate increases with temperature, whereas at low temperatures, the SO phonon scattering rate is not significant. Similarly, it can be concluded from Figure 7 that

(24) $$1/\tau_{Polaron}\left(WS_{2}\right)>1/\tau_{Polaron}\left(W{Se}_{2}\right)>1/\tau_{Polaron}\left(MoS_{2}\right)>1/\tau_{Polaron}\left(Mo{Se}_{2}\right).$$

From Figure 7, we can also observe that the SO phonon scattering rate in ML TMDCs depends strongly on the dielectric constant of the polar substrate, as follows:

(25) $$1/\tau_{Polaron}\left(MLTMDCs/SiC\right)>1/\tau_{Polaron}\left(MLTMDCs/hBN\right)$$

Finally, by choosing a suitable dielectric as a substrate, one can achieve the highest SO phonon scattering rate in ML TMDCs.

To experimentally test the electron–surface optical phonon (SOP) interaction in transition metal dichalcogenides (TMDCs) deposited on silicon carbide or hexagonal boron nitride dielectric substrates, we propose several experiments, such as Photoluminescence (PL) Spectroscopy, Raman spectroscopy, Time-Resolved Photoluminescence (TRPL), Electroluminescence (EL) Spectroscopy, and Angle-Resolved Photoemission Spectroscopy (ARPES) [22,47,48,49,50].

Our theoretical findings reveal that the coupling between surface optical phonons (SOP) and monolayer TMDCs is significantly influenced by the choice of polar substrate and has promising implications for advancing 2D material-based optoelectronic devices. This coupling between electronic sub-levels in TMDCs and surface polar vibration modes on polar substrates not only leads to Rabi splitting but also strengthens electronic interactions with surface phonons. These enhanced interactions open several pathways for optimizing device performance across various applications.

A key insight is that strong resonant coupling between TMDC electrons and surface optical phonons can reduce nonradiative recombination by channeling energy into radiative decay pathways. This boost in photoluminescence efficiency is especially valuable for light-emitting devices, such as LEDs, where maximizing photon emission is crucial. By selecting substrates that induce greater Rabi splitting, such as SiC or hBN, designers can achieve brighter, more efficient emitters.

Furthermore, our findings show that stronger polarization fields at the TMDC–substrate interface lead to increased polaronic optical strength and higher scattering rates. This polarization effect, which can be adjusted by selecting specific polar substrates, has significant implications for carrier mobility. For example, high-dielectric substrates like SiC can enhance mobility by modulating electron–phonon interactions, a result corroborated by Sanjay Gopalan et al. [24]. These insights can inform the selection of substrate materials for engineering high-mobility TMDC transistors, essential for low-power, high-speed electronic devices.

Additionally, coupling in SOP modes creates polaronic states that influence energy relaxation dynamics in TMDCs. By tuning the SOP interaction strength through substrate choice, we can control the rate and pathways of phonon-assisted hot carrier relaxation, which is relevant for applications like photovoltaics and photodetectors. The ability to manage energy dissipation could result in devices with improved efficiency and faster response times.

Our study also highlights that the resonant coupling between TMDC electrons and surface phonons can enhance Raman scattering processes. This enhancement aligns with the experimental findings by Colin M. Chow et al. [22] and can be leveraged in devices that require precise optical control, such as tunable filters, sensors, or Raman spectroscopy applications, as previously demonstrated by Bastian Miller et al. [23].

Overall, these potential applications demonstrate that a deep understanding of substrate-dependent SOP coupling provides a strong foundation for tailoring and improving the optical and electronic properties of monolayer TMDC-based devices. This foundational insight into SOP coupling can guide both material and substrate selection for specific functionalities across various optoelectronic applications.

## 5. Conclusions

In conclusion, our results indicate that the SOP coupling in ML TMDCs depends strongly upon the polar substrate. Moreover, the resonant coupling between electronic sub-levels and surface polar vibration modes leads to the Rabi splitting of electron levels in single-layer TMDCs. Hence, the polarization field induces robust resonant mixing between electronic states and surface vibration modes when their energies become comparable. This resonant coupling has the potential to diminish the probability of nonradiative recombination processes and enhance the efficiency of photoluminescence (PL). Finally, we demonstrate that the highest polarization field created near the interface leads to the highest polaronic OS and the enhancement of SO phonon scattering rates in ML TMDCs. Thus, polaronic OS and polaronic scattering rates depend strongly upon the choice of the polar substrate. The presence of polaronic states may yield significant implications for energy relaxation in ML TMDCs. Moreover, the interaction between electrons and surface optical phonons is a crucial factor influencing the physical characteristics of 2D semiconductors, particularly influencing processes like phonon-assisted hot carrier relaxation.

## Figures and Tables

**Table 1:** The parameters for the surface polar phonon scattering of ML TMDCs on the SiC and hBN polar substrates.

	${SiC\;}^{a}$	${hBN\;}^{b}$
$\varepsilon_{0}$	9.7	5.09
$\varepsilon_{\infty }$	6.5	4.1
$\hbar \omega_{SO}\;\left(\mathrm{meV}\right)$	116.0	101.7
$\hbar \omega_{LO}\;\left(\mathrm{meV}\right)$	123.2	103.7
$F_{\nu }^{2}\;\;\left(\mathrm{meV}\right)$	0.735	0.258

^a^ References [38,39]. ^b^ References [39,40].

**Table 2:** The band gap, effective electron mass and internal distance between the TMDCs and polar substrates.

	${{WS}_{2}}^{a,c}$	${{WSe}_{2}}^{b,c}$	${{MoS}_{2}}^{a,c}$	${{MoSe}_{2}}^{a,c}$
$E_{g}\left(\mathrm{eV}\right)$	2.24	2.37	2.31	2.13
$m_{e}\left(m_{0}\right)$	0.31	0.34	0.45	0.53
$z_{0}\left(Å\right)$	6.06	6.44	6.04	6.45

^a^ Reference [41]. ^b^ Reference [42]. ^c^ Reference [43].

**Table 3:** The crossing values $\left(k\right)$ of the noninteracting states $\left|\psi_{k},1q\rangle \;\right.and\;\left|\psi_{k+q},0q\rangle \right.$ in the ML $WS_{2},WSe_{2},MoS_{2},and\;MoSe_{2}$ for both the SiC and hBN polar substrates.

	The Noninteracting Crossing Levels in the Case of SiC	The Noninteracting Crossing Levels in the Case of hBN
${WS}_{2}$	$k\sim 2.2\;{nm}^{-1}$	$k\sim 2.85\;{nm}^{-1}$
${WSe}_{2}$	$k\sim 2.7{\;nm}^{-1}$	$k\sim 3.45\;{nm}^{-1}$
${MoS}_{2}$	$k\sim 4.25\;{nm}^{-1}$	$k\sim 4.95\;{nm}^{-1}$
${MoSe}_{2}$	$k\sim 3.1\;{nm}^{-1}$	$k\sim 3.9{nm}^{-1}$

**Table 4:** The Rabi splitting of the electron levels in the ML $WS_{2},WSe_{2},MoS_{2},and\;MoSe_{2}$ for both the SiC and hBN polar substrates.

	Rabi Splitting in the Case of SiC	Rabi Splitting in the Case of hBN
${WS}_{2}$	94 meV	70 meV
${WSe}_{2}$	96 meV	72 meV
${MOS}_{2}$	70 meV	54 meV
${MOSe}_{2}$	63 meV	47 meV

## Data Availability

The data presented in this study are available on request from the corresponding author. The data are not publicly available due to privacy issues.

## References

[1] Wang Z., Nie Y., Ou H., Chen D., Cen Y., Liu J., Wu D., Hong G., Li B., Xing G. (2023). Electronic and Optoelectronic Monolayer WSe2 Devices via Transfer-Free Fabrication Method. Nanomaterials.

[2] Ahmed T., Zha J., Lin K.K.H., Kuo H.-C., Tan C., Lin D.H. (2023). Bright and Efficient Light-Emitting Devices Based on 2D Transition Metal Dichalcogenides. Adv. Mater..

[3] Joseph S., Mohan J., Lakshmy S., Thomas S., Chakraborty B., Thomas S., Kalarikkal N. (2023). A review of the synthesis, properties, and applications of 2D transition metal dichalcogenides and their heterostructures. Mater. Chem. Phys..

[4] Lau C.S., Chee J.Y., Cao L., Ooi Z.-E., Tong S.W., Bosman M., Bussolotti F., Deng T., Wu G., Yang S.-W. (2022). Gate-Defined Quantum Confinement in CVD 2D WS2. Adv. Mater..

[5] Zhang L., Ni R., Zhou Y. (2023). Controlling quantum phases of electrons and excitons in moiré superlattices. J. Appl. Phys..

[6] Ardizzone V., De Marco L., De Giorgi M., Dominici L., Ballarini D., Sanvitto D. (2019). Emerging 2D materials for room-temperature polaritonics. Nanophotonics.

[7] Xie K., Li X., Cao T. (2021). Theory and Ab Initio Calculation of Optically Excited States—Recent Advances in 2D Materials. Adv. Mater..

[8] Palummo M., Bernardi M., Grossman J.C. (2015). Exciton Radiative Lifetimes in Two-Dimensional Transition Metal Dichalcogenides. Nano Lett..

[9] Qiu D.Y., Cao T., Steven G. (2015). Louie, Nonanalyticity, Valley Quantum Phases, and Lightlike Exciton Dispersion in Monolayer Transition Metal Dichalcogenides: Theory and First-Principles Calculations. Phys. Rev. Lett..

[10] Huang C.-C., Wang H., Cao Y., Weatherby E., Richheimer F., Wood S., Jiang S., Wei D., Dong Y., Lu X. (2022). Facilitating Uniform Large-Scale MoS2, WS2 Monolayers, and Their Heterostructures through van der Waals Epitaxy. ACS Appl. Mater. Interfaces.

[11] Wada N., Pu J., Takaguchi Y., Zhang W., Liu Z., Endo T., Irisawa T., Matsuda K., Miyauchi Y., Takenobu T. (2022). Efficient and Chiral Electroluminescence from In-Plane Heterostructure of Transition Metal Dichalcogenide Monolayers. Adv. Funct. Mater..

[12] Robert C., Han B., Kapuscinski P., Delhomme A., Faugeras C., Amand T., Molas M.R., Bartos M., Watanabe K., Taniguchi T. (2020). Measurement of the spin-forbidden dark excitons in MoS2 and MoSe2 monolayers. Nat. Commun..

[13] Carrascoso F., Li H., Frisenda R., Castellanos-Gomez A. (2021). Strain engineering in single-, bi- and tri-layer MoS2, MoSe2, WS2 and WSe2. Nano Res..

[14] Mahdouani M. (2017). Investigation of the electron-surface phonon interaction effects in graphene on a substrate made of polar materials. PHYSE.

[15] Mahdouani M., Gardelis S., Bourguiga R. (2018). The effect of Si impurities on the transport properties and the electron-surface phonon interaction in single layer graphene deposited on polar substrates. Phys. B Condens. Matter.

[16] Mahdouani M., Bourguiga R. (2017). Auger and carrier-surface phonon interaction processes in graphene on a substrate made of polar materials. Superlattices Microstruct..

[17] Perebeinos V., Avouris P. (2010). Inelastic scattering and current saturation in graphene. Phys. Rev. B.

[18] Mahdouani M., Bourguiga R., Jaziri S. (2008). Polaronic states in Si nanocrystals embedded in SiO_2_ matrix. Physica E.

[19] Mahdouani M., Gardelis S., Nassiopoulou A.G. (2011). Role of surface vibration modes in Si nanocrystals within light emitting porous Si at the strong confinement regime. J. Appl. Phys..

[20] Gardelis S., Nassiopoulou A.G., Mahdouani M., Bourguiga R., Jaziri S. (2009). Enhancement and red shift of photoluminescence (PL) of fresh porous Si under prolonged laser irradiation or ageing: Role of surface vibration modes. Physica E.

[21] Paul S., Karak S., Talukdar S., Negi D., Saha S. (2024). Influence of Edges and Interlayer Electron–phonon Coupling in WS2/h-BN Heterostructure. ACS Appl. Mater. Interfaces.

[22] Chow C.M., Yu H., Jones A.M., Yan J., Mandrus D.G., Taniguchi T., Watanabe K., Yao W., Xu X. (2017). Unusual Exciton–Phonon Interactions at van der Waals Engineered Interfaces. Nano Lett..

[23] Miller B., Lindlau J., Bommert M., Neumann A., Yamaguchi H., Högele A. (2019). Tuning the Fröhlich exciton-phonon scattering in monolayer MoS. Nat. Commun..

[24] Gopalan S., Van de Put M.L., Gaddemane G., Fischetti M.V. (2022). Theoretical Study of Electronic Transport in Two-Dimensional Transition Metal Dichalcogenides: Effects of the Dielectric Environment. Phys. Rev. Appl..

[25] Glazov M.M., Ivchenko E.L. (2021). Valley Orientation of Electrons and Excitons in Atomically Thin Transition Metal Dichalcogenide Monolayers. JETP Lett..

[26] Durnev M.V., Glazov M.M. (2018). Excitons and trions in two-dimensional semiconductors based on transition metal dichalcogenides. Phys.–Uspekhi.

[27] Kormanyos A., Burkard G., Gmitra M., Fabian J., Zolyomi V., Drummond N.D., Fal’ko V. (2015). k·p theory for two-dimensional transition metal dichalcogenide semiconductors. 2D Mater..

[28] Antonius G., Louie S.G. (2022). Theory of exciton-phonon coupling. Phys. Rev. B.

[29] Sohier T., Calandra M., Mauri F. (2016). Two-dimensional Fröhlich interaction in transition-metal dichalcogenide monolayers: Theoretical modeling and first-principles calculations. Phys. Rev. B.

[30] Wang Y., He C., Tan Q., Tang Z., Huang L., Liu L., Yin J., Wang X., Pan A. (2023). Exciton–phonon coupling in two-dimensional layered (BA)2PbI4 perovskite microplates. RSC Adv..

[31] Ma J., Xu D., Hu R., Luodoi X. (2020). Examining two-dimensional Fröhlich model and enhancing the electron mobility of monolayer InSe by dielectric engineering. J. Appl. Phys..

[32] Hinsche N.F., Thygesen K.S. (2017). Electron–phonon interaction and transport properties of metallic bulk and monolayer transition metal dichalcogenide TaS2. 2D Mater..

[33] Xiao Y., Li Z.Q., Wang Z.W. (2017). Polaron effect on the bandgap modulation in monolayer transition metal dichalcogenides. J. Phys. Cond. Matter.

[34] Nguepnang J.V., Kenfack C., Kenfack A., Fobasso M.F.C., Sun Y. (2021). Optical signature of bipolaron in monolayer transition metal dichalcogenides: All coupling approach. Opt. Quantum Electron..

[35] Devreese J.T., Huybrechts W., Lemmeks L. (1971). On the optical absorption of free polarons at weak coupling. Phys. Status Solidi.

[36] Mafra D.L., Araujo P.T. (2014). Intra- and Interlayer Electron-Phonon Interactions in 12/12C and 12/13C BiLayer Graphene. Appl. Sci..

[37] Wang S.Q., Mahan G.D. (1972). Electron Scattering from Surface Excitations. Phys. Rev. B.

[38] Schiefele J., Sols F., Guinea F. (2012). Temperature dependence of the conductivity of graphene on boron nitride. Phys. Rev. B.

[39] Geick R., Perry C.H., Rupprecht G. (1966). Normal Modes in Hexagonal Boron Nitride. Phys. Rev..

[40] Rozhkov A.V., Nori F. (2010). Exact wave functions for an electron on a graphene triangular quantum dot. Phys. Rev. B.

[41] Han B., Robert C., Courtade E., Manca M., Shree S., Amand T., Renucci P., Taniguchi T., Watanabe K., Marie X. (2018). Exciton States in Monolayer MoSe_2_ and MoTe_2_ Probed by Up conversion Spectroscopy. Phys. Rev. X.

[42] Wang G., Gerber I.C., Bouet L., Lagarde D., Balocchi A., Vidal M., Palleau E., Amand T., Marie X., Urbaszek B. (2015). Exciton states in monolayer MoSe2: Impact on interband transitions. 2D Mater..

[43] Laturia A., Van de Put M.L., Vandenberghe W.G. (2018). Dielectric properties of hexagonal boron nitride and transition metal dichalcogenides: From monolayer to bulk. 2D Mater. Appl..

[44] Benedict L.X., Louie S.G., Cohen M.L. (1995). Static polarizabilities of single-wall carbon nanotubes. Phys. Rev. B.

[45] Hwang E.H., Sarma S.D. (2013). Surface polar optical phonon interaction induced many-body effects and hot-electron relaxation in graphene. Phys. Rev.B.

[46] Mahdouani M., Zalfani M., Bourguiga R., Su B.-L. (2019). Radiative and non radiative recombinations study in the novel nanocomposites BiVO_4_/3DOM-TiO_2_, ZnO/3DOM-TiO_2_ and BiVO_4_/3DOM-ZnO: Application to the photocatalysis. Physica E.

[47] Chang Y.-C., Chan Y.-C., Das B., Syue J.-F., Hu H.-C., Lan Y.-W., Lu T.-H. (2024). Distinctive characteristics of exciton-phonon interactions in optically driven MoS. Phys. Rev. Mater..

[48] Nguepnang J.V., Kenfack-Sadem C., Kenfack-Jiotsa A., Guimapi C., Fotue A.J., Merad A.E. (2021). Electron–phonon coupling contribution on the optical absorption and the dynamic of exciton-polaron in monolayer Transition Metal Dichalcogenides. Opt. Quantum Electron..

[49] Lai J.-M., Xie Y.-R., Zhang J. (2021). Detection of electron-phonon coupling in two-dimensional materials by light scattering. Nano Res..

[50] Jiang Y., Chen S., Zheng W., Zheng B., Pan A. (2021). Interlayer exciton formation, relaxation, and transport in TMD van der Waals heterostructures. Light Sci. Appl..

